# I-SCREEN: Development of an AI-based infrastructure for community-wide screening and prediction of progression in age-related macular degeneration providing accessible shared care

**DOI:** 10.1038/s41433-026-04487-0

**Published:** 2026-05-05

**Authors:** Marie Louise Enzendorfer, Gregor S. Reiter, Hrvoje Bogunović, Sophie Riedl, Julia Mai, Johannes Schrittwieser, Christoph Stapf, Virginia Mares, Matjaž Mihelčič, Julie-Anne Little, Polona Jaki-Mekjavić, Daniel Barthelmes, Katja Hatz, Javier Zarranz-Ventura, Catherine Creuzot-Garcher, Ruth Hogg, Amir Sadeghipour, Ursula Schmidt-Erfurth

**Affiliations:** 1 Laboratory for Ophthalmic Image Analysis (OPTIMA), Medical University of Vienna, Vienna, Austria; 2 Department of Ophthalmology and Optometry, Medical University of Vienna, Vienna, Austria; 3 Institute of Artificial Intelligence, Center for Medical Data Science, Medical University of Vienna, Vienna, Austria; 4 European Council of Optometry and Optics, Adligenswil, Switzerland; 5 Centre for Optometry and Vision Science, School of Biomedical Sciences, Ulster University, Coleraine, UK; 6 Department of Ophthalmology, University Medical Centre Ljubljana, Ljubljana, Slovenia; 7 Faculty of Medicine, University of Ljubljana, Ljubljana, Slovenia; 8 Jozef Stefan Institute, Ljubljana, Slovenia; 9 Department of Ophthalmology, University Hospital Zurich and University of Zurich, Zurich, Switzerland; 10 Vista Augenklinik Binningen, Binningen, Switzerland; 11 Faculty of Medicine, University of Basel, Basel, Switzerland; 12 Hospital Clínic de Barcelona, Universitat de Barcelona, Barcelona, Spain; 13 Fundació de Recerca Clínic Barcelona-Institut d’Investigacions Biomèdiques August Pi i Sunyer (FCRB-IDIBAPS), Barcelona, Spain; 14 University Bourgogne Europe, Dijon, France; 15 Ophthalmology Department, Dijon University Hospital, Dijon, France; 16 Centre for Public Health, School of Medicine, Dentistry, and Biomedical Science, Queen’s University Belfast, Belfast, UK; 17 RetInSight GmbH, Vienna, Austria; 18 EURICE – European Research and Project Office GmbH, St. Ingbert, Germany; 19 Research and Innovation Services-RISE d.o.o., Zagreb, Croatia

**Keywords:** Medical imaging, Biomarkers

## Abstract

**Objectives:**

This work describes the design and methodological framework of the I-SCREEN project, which aims to develop an artificial intelligence (AI)-based infrastructure utilising optical coherence tomography (OCT) for early detection of AMD and assessment of progression risk.

**Methods:**

The pan-European project is conducted across clinics and optometry/optician practices in six European countries. I-SCREEN encompasses seven work packages covering community-based AMD identification, clinical follow-up, AI development and project dissemination. Three interconnected clinical studies are carried out by optometry/optician practices (PYRENEES) and ophthalmology clinics (SUDETES and APENNINES).

**Results:**

The PYRENEES study is a prospective, cross-sectional study evaluating the feasibility of detecting subclinical AMD in optometry/optician practices under ophthalmologist supervision via telemedicine. A robust screening network comprising 28 community-based optometry/optician practices and 7 ophthalmology clinics has been established. Patients with suspected non-neovascular AMD are referred to partnered clinics. In the hospital setting, patients with early or intermediate AMD are followed in the longitudinal SUDETES study, while patients with non-foveal geographic atrophy are invited to take part in the APENNINES study. Data obtained inform AI development for community-based AMD detection and monitoring. Predictive modelling will further enable personalised risk assessments.

**Conclusions:**

I-SCREEN brings together multidisciplinary experts across Europe to establish an AI-driven shared care model for AMD detection and monitoring. By combining high-quality OCT imaging from community practices with longitudinal clinical studies, the initiative provides novel insights into early AMD progression and  establishes a foundation for innovative AI-based detection and prediction throughout the real-world population.

## Introduction

Age-related macular degeneration (AMD) is one the most common causes of vision loss, affecting almost 20% of the older population and more than 15 million individuals in Europe [1]. The disease progresses slowly from early and intermediate AMD (iAMD) - characterised by the accumulation of extracellular lipid-rich deposits known as drusen in the macula together with progressive alteration of the retinal pigment epithelium (RPE) and neurosensory retina - to advanced AMD with common complications of geographic atrophy (GA) and macular neovascularisation (MNV). At its early and intermediate stages, patients often remain asymptomatic as atrophic lesions develop outside the central retina, leaving the fovea responsible for central vision intact. AMD is therefore largely underdiagnosed until substantial irreversible vision impairment occurs, thus limiting the window for effective treatment [2].

With the global population aging, the prevalence of age-related disease, such as AMD, is expected to increase at an unprecedented rate [3]. Thus, novel strategies that provide accessible and timely care to patients and efficient screening for early AMD detection are imperative. With advances in optical coherence tomography (OCT) technology, spectral-domain (SD)-OCT has become a gold standard in the management of AMD, facilitating detection of AMD even at the earliest pathologic changes, due to the high-resolution and three-dimensional (3D) nature of the images provided [4]. The practical and non-invasive technology has made it the most frequent diagnostic measure for macular pathologies and essential for monitoring of disease progression. In parallel, the integration of OCT imaging in community-based eye care settings is becoming commonplace in the UK and Europe [5, 6].

Moreover, with the advent of deep learning (DL), artificial intelligence (AI)-based tools for medical image analysis have developed rapidly. Especially in the field of ophthalmology, where large datasets are available, significant advances have been made and AI approaches have shown to be successful in objectively identifying early pathognomonic changes to support the decision-making process for clinical ophthalmologists [7]. Importantly, DL-based models present an opportunity for risk prediction, using imaging, demographic and often genetic data as an input to identify an individual’s risk for disease progression to more severe stages of the disease [8–10].

The use of AI for OCT image analysis serves as an opportunity to bring high-precision screening of AMD directly to the wider community, enabling access to early diagnosis and timely treatment for a high-risk population that does not yet present symptoms of AMD. The I-SCREEN consortium is a pan-European collaboration between primary eye care professionals, specialised clinics and computer science experts to create an AI-based infrastructure for screening and prediction of progression in AMD, enabling standardised shared care. Herein, we report on the overall project framework and clinical protocol design being implemented over a four-year project period. Given the size and complexity of the initiative it is essential to document the combined efforts, methodological steps and prior work underpinning the I-SCREEN infrastructure. This report, therefore, provides a foundational reference for subsequent publications presenting the project’s outcomes.

## Methods and design

### Consortium structure

The I-SCREEN consortium is funded by the Horizon European Innovation Council (EIC) 2023 Pathfinder Call (No. 101130093) under the name “I(eye)-SCREEN: A real-world AI-based infrastructure for screening and prediction of progression in age-related macular degeneration (AMD) providing accessible shared care”. To optimally achieve the goals and objectives of the interdisciplinary consortium, I-SCREEN is subdivided into seven work packages (WPs): Observational cohorts of patients with intermediate/atrophic AMD (WP1), Artificial Intelligence science for Retinal Imaging (WP2), Image collection and screening algorithm for intermediate/atrophic AMD detection by a cloud-based platform (WP3), AI-based detection of AMD in community-based low-cost OCT imaging (WP4), Project management and scientific coordination (WP5), Innovation Management (WP6), and Ethics requirements (WP7).

The European Council of Optometry and Optics (ECOO) has built a network of community-based optometry/optician practices across Europe, offering extensive screening of individuals in the AMD risk population within the PYRENEES study (WP4, NCT06465212), under the clinical oversight of board-certified ophthalmologists. Participation of primary eye care specialists provides unique real-world data and a validation set for the development of an efficient AI-based screening system for AMD. Longitudinal clinical data to study the progression of identified disease is collected by seven clinical sites in six European countries (Table 1) within two observational studies, SUDETES for conversion (WP1, NCT06351670) and APENNINES for progression of non-neovascular AMD (WP1, NCT06351657). A cloud-based platform for data collection has been developed by RetInSight, a medium-sized enterprise with established AI-based clinical decision support expertise. A computer science group focusing on AI development for OCT analysis, based at the Institute of Artificial Intelligence at the Medical University of Vienna, develops AI methods for biomarker research and predictive modelling of disease progression. Moreover, this work addresses the challenge of transferring AI models across scans of high-budget and low-budget OCT devices and from the clinical setting to a community-based scenario. To provide efficient dissemination of project results to be applied in the primary care setting and a successful intellectual property (IP) strategy, an innovation office, EURICE, is supporting all collaborative activities within the consortium. An independent ethics advisory board, consisting of multidisciplinary experts across Europe, addresses any challenges related to the complexity of the project, AI development and its implementation in real-world healthcare.

**Table 1:** Overview of all participating sites in the I-SCREEN shared care network.

Country	Clinical partner	Paired optometry/optician practice	Location
Austria	Medical University of Vienna	AUGE & SEHEN	Vienna
		Dr. Gumpelmayer Augenoptik	Linz
		Hartmann Wien	Vienna
France	Centre Hospitalier Reg Universitaire Dijon	Centre Hospitalier Reg Univeritaire Dijon general walk-in clinic	Dijon
Slovenia	University Medical Centre Ljubljana	Igor Vogrič s.p	Idrija
		OKE d.o.o.	Golnik
		Optika Mesec Bled	Bled
Spain	Hospital Clínic de Barcelona	Boldú Optic	Tarragona
		Facultat d'Òptica i Optometria de Terrassa	Terrassa
		FEDERÒPTICS	Lleida
		Natural Optics Balaguer	Lleida
		Òptipunt	Girona
Switzerland	University Hospital Zürich	Augentreff	Lenzburg
		Frey Peter Gmbh	Frick
		Herzog Optik	Cham
		SCHWARZ Optik GmbH	Heiden
		Sehzentrum Zürich	Zürich
		Strebel Optik	Wohlen
Switzerland	VISTA Eye Clinic Binningen	Institute of Optometry, FHNW	Olten
		Iseli Optik	Basel
		Kohler Optik AG	Oensingen
United Kingdom	Queen’s University Belfast	EK Eyewear	Belfast
		Faith Donaldson	Newry
		Graeme Black Eyecare	Cookstown
		Harris Rundle	Belfast
		I-Care	Portrush
		Jill McKeown Opticians	Banbridge
		Judith Long Opticians	Clough
		RA Glass	Belfast

Overview of all participating sites of the I-SCREEN shared-care network and PYRENEES community-based screening.

The project is regularly reviewed by an external review committee appointed by the European Commission. This committee consists of AI, retina and innovation experts and provides consistent monitoring of the project status and outcomes.

### Protocol development

Study protocols for the three clinical studies were designed to address the challenge of real-world data availability for AI development and together form the framework for a shared care strategy for screening and monitoring of AMD in a community setting and at an affordable scale. The design, sample size and endpoints were decided based on a comprehensive review of the available literature up to October 2023.

## Results

### PYRENEES: community-based cross-sectional screening at optometry/optician practices

#### Study design

The PYRENEES study addresses the need for large-scale data of a real-world population before and beyond established ophthalmology to provide information on preclinical disease and represents the first study of its kind beyond a conventional clinical setting. The prospective cross-sectional study provides community-based screening using the Maestro OCT device (Topcon, Tokyo, Japan) at optometry/optician practices, identifying individuals with non-vision compromising AMD otherwise remaining undiagnosed. A robust screening network has been established, comprising 28 community-based optometry/optician practices and 7 partnering clinics in close proximity, contributing to the screening for part or the full duration of the PYRENEES study. For each clinic, 3–8 optometry practices are included, with capacity for other optometry practices to still join the project. The median distance between clinic and optometry practice is 40.2 km (IQR: 5.2–98.0 km), allowing for commutes under 2 h.

Allcomers suspected at risk for AMD, 55–99 years of age, and with preserved visual function (<0.3 logMAR) are invited to participate in screening for AMD at optometrist/optician practices performing regular primary eye care procedures in real-world routine (Table 1). In addition to a colour fundus photograph (CFP), OCT imaging is acquired for each eye using a standardised protocol comprising a 512 × 128 volume scan over a 6.0 × 6.0 mm field of view. The acquired images are uploaded to a protected data collection platform (cAIre Direct, RetInSight, Vienna, Austria) and independently evaluated by a board-certified ophthalmologist (telemedicine doctor). A full informed consent to data transfer must be provided by the participant prior to any data upload.

**Fig. 1:**
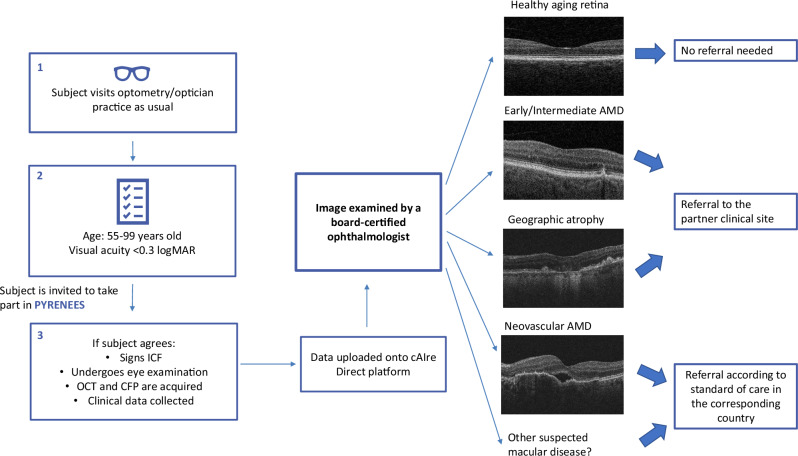
Design of the community-based screening for AMD within the PYRENEES study. ICF Informed consent form, OCT Optical Coherence Tomography, CFP Colour Fundus Photography, AMD Age-related macular degeneration.

Individualised referral recommendations are given within 5 business days based on predefined criteria (Table 2*)*. For each uploaded OCT volume, all 128 B-scans are systematically reviewed for the presence of drusen >63 μm in size and/or complete RPE and outer retinal atrophy (cRORA), determined by a threshold of >250 µm in diameter [11, 12]. Detection of these markers is considered indicative of non-neovascular AMD and triggers referral to the partnering ophthalmology clinic. Measurements are performed using the built-in measurement tools of the cAIre Direct platform, ensuring standardised assessment across all data. Participants referred to the partnering clinical site (Table 1) are enrolled into longitudinal clinical studies, SUDETES or APENNINES, if the inclusion/exclusion criteria for the clinical studies are met. A simplified summary of the study design is shown in Fig. 1.

**Table 2:** Referral recommendations provided through telemedicine based on optical coherence tomography (OCT) image grading.

Criteria based on OCT image grading	Referral recommendation
Healthy aging retina including drupelets <63 μm	No referral needed.
Early/Intermediate AMD defined by drusen >63 µm with or without pigmentary abnormalities [11]	Direct referral within 12 weeks to the partnering clinical site.
Late atrophic AMD defined by a region of hypertransmission, retinal pigment epithelium and photoreceptor attenuation of >250 µm [12]	Direct referral within 12 weeks to the partnering clinical site.
Active neovascular AMD defined by outer retinal alterations and intraretinal fluid (IRF), subretinal fluid (SRF) or pigment epithelial detachment (PED)	Urgent referral to the patient’s ophthalmologist within 0–14 days. Participant will be treated according to the standard of care in the corresponding country.
Suspected RVO, CCS, DME	Urgent referral to the patient’s ophthalmologist within 0–14 days. Participant will be treated according to the standard of care in the corresponding country.
Suspected other pathology	Recommendation of referral based on the individual case and severity of the underlying suspected disease.

PYRENEES has been approved by the Ethics Committee of the Medical University of Vienna (EC: 1318/2024), as well as central and institutional ethics committees in all participating countries. The study has been registered on clinicaltrials.gov (NCT06465212). The study is conducted in accordance with the Declaration of Helsinki and adheres to the principles of Good Clinical Practice. The first subject was screened for AMD in January 2025. An 18-month timeframe for recruitment is planned for the study. As of end of February 2026, over 2,300 participants have been imaged. Around 20% of screened individuals have been identified as having signs of early or iAMD or non-vision affecting geographic atrophy (GA) and have consequently been referred to the partner clinic for a 2-year monitoring.

#### Study outcomes

PYRENEES assesses the identification of different AMD stages on OCT images through the unique network based on OCT imaging and telemedicine, establishing a real-world cohort of individuals with AMD without clinically relevant visual impairment and outside of ophthalmologists’ care. The proportion and distribution of early AMD, iAMD and early GA cases within this real-world cohort can be determined. As a secondary outcome, an unprecedented characterisation of the structural morphology of this community-based cohort will be performed, analysing a previously unknown spectrum of OCT-based biomarkers of AMD. Biomarker categories of interest include drusen, hyperreflective foci, subretinal drusenoid deposits, loss of outer retinal layers and retinal fluid.

### SUDETES and APENNINES: longitudinal, observational studies of patients with early/intermediate AMD or non-foveal GA

#### Study design

Two 24-month prospective cohort studies allow for the collection of longitudinal data for the analysis of AMD disease progression. SUDETES includes 500 patients with early or iAMD, defined by the presence of drusen >63 µm and/or any definite hyper- or hypopigmentary abnormalities [11], allowing for observation of disease progression and conversion from the intermediate to the late-stage of AMD (GA or neovascular AMD). APENNINES monitors 200 patients with non-foveal GA, defined by the presence of complete RPE and outer retinal atrophy >250 µm in diameter (cRORA) [12] and a BCVA ≥ 20/40 (≤0.3 logMAR). Both longitudinal studies include subjects from the cross-sectional PYRENEES study matching the protocol criteria, as well as patients conforming with the same protocol criteria from untreated outpatient routine at the participating clinics. If both eyes are eligible, two eyes can be included in the respective study. Both studies are described in synergy as they include identical study procedures and are conducted at the same seven clinics. Full inclusion and exclusion criteria are provided within the respective ClinicalTrials.gov registries (NCT06351670, NCT06351657) [13, 14]. All patients provide written informed consent in the language of the country of the clinic. The studies are conducted in accordance with the Declaration of Helsinki and adhere to the principles of Good Clinical Practice.

Patients included in the studies are followed at 6-month intervals over 24 months. At each visit, a standardised eye examination, imaging and functional tests are carried out (Table 3). OCT imaging is performed with two different devices at every clinical site: the Spectralis HRA + OCT (Heidelberg Engineering, Heidelberg, Germany) representing a clinically and scientifically used prototype and the Maestro2 (Topcon, Tokyo, Japan) device as a low-budget solution more widely used in community-based eye care settings. At each visit, the Spectralis OCT is automatically analysed by the MDR-certified GA monitor (RetInSight, Vienna, Austria) [15], allowing for AI-based quantification of the photoreceptors, i.e. the ellipsoid zone (EZ) layer, and RPE layer in real-time. In SUDETES, the MDR-certified Fluid Monitor (RetInSight, Vienna, Austria) is used additionally for early real-time fluid detection in converters. After each visit, an AI Questionnaire is completed by the clinician to independently assess the performance and assistive capability of automated image analysis.

**Table 3:** Overview of the procedures within the SUDETES and APENNINES studies.

Assessment/Activity	Visit 1	Visit 2	Visit 3	Visit 4	Visit 5
	Baseline	Month 6^+^	Month 12^+^	Month 18^+^	Month 24^+^
		+/−2 Weeks	+/−2 Weeks	+/−2 Weeks	+/−2 Weeks
Informed consent	x				
Medical history/adverse events	x	x	x	x	x
BCVA	x	x	x	x	x
OCT Heidelberg Engineering Spectralis	x	x	x	x	x
OCT Topcon Maestro2	x	x	x	x	x
OCT angiography^a^	x	x	x	x	x
CFP^a^	x	x	x	x	x
FAF^a^	x	x	x	x	x
Clinical examination	x	x	x	x	x
NEI-VFQ	x		x		x
QS on AI use	x	x	x	x	x

^a^If available at study site.

*BCVA* best-corrected visual acuity, *OCT* optical coherence tomography, *CFP* colour fundus photography, *FAF* fundus autofluorescence, *NEI-VFQ* National Eye Institute – Visual Function Questionnaire, *QS* questionnaire.

Functional tests include best-corrected visual acuity (BCVA) assessments using a standard ETDRS chart at 4 meters at every visit, as well as the National Eye Institute – Visual Function Questionnaire 25 (NEI-VFQ) every twelve months.

Both studies have been approved by the Ethics Committee of the Medical University of Vienna (EC: 1109/2024 and 1088/2024), as well as by central and institutional ethics committees in all participating countries. Following availability of imaging devices, ethics approvals, and contracting, the first patients were enrolled at different time points at the participating clinics. The first patient was enrolled in February 2025. As of end of February 2026, over 400 participants have been enrolled across the seven clinical sites for the SUDETES study. For APENNINES, 90 participants have been enrolled.

#### Sample size

For SUDETES, sample size calculations are based on the requirements for the development of a predictive model that will predict the risk of conversion from early/intermediate to late AMD. Based on previous studies, conversion from intermediate to late AMD is predicted to occur at a 20% rate [16]. A formula proposed by Newcombe, derived from a nonparametric approach to avoid assumptions about the underlying distribution of predictors, was used to estimate the minimum sample size [17]. A C-statistic of 0.8 is expected for the planned predictive model based on prior work and available literature [10]. For a targeted confidence interval of 0.1, 471 patients would have to be enrolled. Taking into consideration that some patients may drop out of the study, a total of 500 iAMD patients for inclusion at baseline was planned.

For the APENNINES study, the sample size was calculated based on previously reported rates of natural GA growth as measured on OCT, with the objective of characterising fast versus slow disease progression using both descriptive analyses and DL-based modelling. Based on previous literature, in the absence of treatment, the average annual square root area of lesion growth is 0.37 mm/year with a standard deviation of 0.20 mm/year [18, 19]. A clinically significant difference from this natural progression speed was defined as a 25% increase in the annual square root area lesion growth rate. This threshold is used to stratify patients into a fast or slow progression group. To detect this clinically significant difference between the fast progression group and the rest of the study population - with a power of 80% and alpha = 0.05, and accounting for patient loss to follow-up, a total sample size of 200 patients in total was determined.

#### Study outcomes

A comprehensive assessment of OCT imaging biomarkers and their association with disease progression will be performed. In SUDETES, the association of imaging biomarkers with disease conversion from early/iAMD to late-stage AMD will be evaluated. In APENNINES, their association with GA lesion growth rate specifically in early asymptomatic GA lesions will be assessed. Biomarkers assessed will include hyperreflective foci, drusen volume, subretinal drusenoid deposits, EZ loss, RPE loss, and thinning as well as loss of outer retinal layers. Measurements of EZ and RPE loss will be provided by AI-based image analysis using the GA Monitor (RetInSight, Vienna, Austria). In SUDETES, the measurement of fluid volumes in eyes which convert to neovascular AMD will be provided by AI-based image analysis using the Fluid Monitor (RetInSight, Vienna, Austria).

The extensive evaluation of imaging features over time will be used to allow for a standardised individual risk assessment independent of the observer setting and training, and will be used for the development of novel AI-based risk prediction models for AMD. In addition, the studies will qualitatively evaluate the benefit and usage of AI-based monitoring of atrophy and fluid by including an AI questionnaire carried out by clinicians.

### Data management by a cloud-based data collection platform

All data is managed in a central web-based data collection platform, cAIre Direct (RetInSight, Vienna, Austria). cAIre Direct provides the consortium throughout primary and secondary eye care professionals with a means of a comprehensive organised data management. The data is stored in ISO27001-certified data centres located in the European Union (EU). All participating study sites are given secure access to the platform for data upload. The collected data will be made available to consortium members upon request and are subject to restrictions in compliance with General Data Protection Regulation (GDPR).

### Development of an AI-based predictive model of community-based early AMD

Predictive models are being developed using the OCT volume data collected within the WP1 longitudinal studies SUDETES and APENNINES, where 20% of patients in each of the two prospective studies will be preserved as hold-out test sets. For training, we also rely on existing retrospective longitudinal datasets of patients with early/intermediate AMD [20] and geographic atrophy [21]. Methods for data-efficient deep learning from longitudinal OCT data are addressed, allowing for prediction of the disease progression at an arbitrary time. Specifically, temporal self-supervised learning methodologies are being developed, integrating a Siamese masked autoencoder (SiamMAE) with temporal encoding to introduce stochasticity and allowing for the interpretation of time-dependent information from patient visits [22]. This is of high importance for minimising the need for data curation by experts and for efficiently exploiting the available unlabelled imaging data. In addition, it tackles the methodological challenges around (i) high-dimensional data representation brought by OCT volumes, (ii) temporality of underlying acquired patient data, and (iii) data-efficient training. Finally, principles of trustworthy AI, such as FUTURE-AI [23] will be followed in all aspects of the AI development, from data collection to AI model implementation.

A non-linear combination of OCT-based imaging biomarkers, previously validated as important prognostic markers, are employed to achieve a high level of predictive performance [24]. The AI-based risk estimation will use a 3D OCT volume from one or multiple prior patient examinations to predict if the individual eye will convert to advanced AMD (MNV or GA) within a defined time and also provide the clinician with a risk score, 0 ≤ *r* < 1.0, inversely related to the time to conversion. For an input already showing GA, a disease mask of the predicted lesion growth at arbitrary time T is defined as output. This process will build on previous work published by Lachinov et al. [25].

To make the developed predictive models accessible in community-based settings, they are adapted for use on the more widely distributed Maestro device used in the primary eye care domain. For this purpose, the WP1 studies, SUDETES and APENNINES, incorporate dual imaging with the Spectralis and Maestro2 at every patient visit. An innovative image domain adaptation technique based on diffusion models [26] will be developed for reconstruction of the Maestro image to adjust similarity to its paired Spectralis image allowing for high-level accuracy of the models on both OCTs.

In addition, demographic factors are being incorporated into all stages of model development to support robust performance across variations in age, sex, and ethnicity, to the extent possible. Nevertheless, potential limitations related to population diversity must be acknowledged, as the study cohort is derived from a European research setting, where ethnic diversity may be limited, and a specific age range of 55–99 years has been defined for all clinical studies. These constraints need to be considered when interpreting the results and in any future real-world deployment of the models.

### Validation of an AI-based screening algorithm for the wider community

An automated screening algorithm based on previous segmentation work will be validated for a real-time identification of individuals with early/intermediate AMD. The algorithm is based on deep learning driven segmentation of relevant OCT biomarkers, specifically drusen, and RPE alteration, allowing for accurate delineation and identification. High focus is put on maintaining the interpretability and sensitivity of the algorithm for its use in general community-settings, considering the use of low-budget OCT imaging devices and the diversity in screening populations. The output of the algorithm will be compared with the gradings of the human expert reference. The accuracy of the automated diagnostic tool will be reported as sensitivity and specificity of diagnosis with 95% confidence intervals.

The algorithm is being developed to run on the cloud-based platform, making automated disease detection readily available for use in community-based eye care settings, thereby offering “next door” screening in a timely and inexpensive manner to the elderly population. Extension of AI-secured diagnostic tools will not only broaden care to communities not reaching clinical care, but also substantially reduce healthcare costs in one of the most prevalent diseases of modern times, i.e. AMD.

## Discussion

A major challenge in current AMD management is delayed diagnosis and a lack of timely treatment, as morphological changes precede symptoms by years, often postponing presentation to ophthalmologists. The EIC-funded I-SCREEN project addresses this gap by developing an AI-based infrastructure for reliable early AMD screening and progression prediction, enabling real-time, automated use in both community and hospital settings to support an efficient and standardised shared care model across Europe.

At the core of I-SCREEN is the integration of AI with OCT imaging. Although various population-based screening programs for retinal diseases – including AMD – have been reported, the majority rely on CFP imaging due to its wider availability [27]. However, previous studies evaluating AMD screening strategies have demonstrated that CFP images often lack the resolution necessary to detect the subtle, early morphological changes characteristic of AMD [28, 29]. In contrast, OCT provides high-resolution cross-sectional images that reveal early pathology often invisible in fundus photographs, supporting its suitability for screening of early AMD [30]. Furthermore, prior work has shown that AI can significantly enhance the interpretation of OCT images, enabling more accurate and scalable identification of individuals at risk of progression, potentially before symptoms emerge [31, 32]. The 3D high-resolution capacity of OCT imaging combined with AI tools allows for detection of subtle changes qualitatively and most importantly quantification of progressive feature alteration.

The breakthrough concept of I-SCREEN is the involvement of primary eye care, i.e. opticians and optometrists across Europe. This professional level typically encounters the individuals relevant for AMD screening, individuals over the age of 55 years becoming dependent on reading glasses or experiencing mild vision disturbance due to dry eye or early cataract. This positions them in an important position for AMD screening, due to their work with individuals in which the disease is still early before sensitive macular structures are irreversibly damaged. Recent evidence has further indicated the benefits and high acceptance of community-based monitoring of AMD by patients and health care professionals in the UK, supporting the feasibility of shifting selected aspects of AMD care outside hospital settings [33]. In recent years, low-budget OCT devices have been adopted by more and more primary eye care professionals as a mean of objective visualisation of retinal pathology. Because diagnosis of the subtle signs of OCT features associated with AMD progression is difficult, with concordance between experts being relatively poor [24], AI-supported shared screening now offers a reliable and efficient pathway for screening and monitoring covering the long-needed enhancement of population-wide care. Simultaneously, over-referral will be avoided by introducing defined criteria for fast progression based on the development of AI predictive tools which recognise those individuals which benefit from clinical observation and eventually intervention.

Collaboration between optometrists/opticians and ophthalmologists is central, though this strategy complements rather than replaces regular ophthalmic examinations, particularly given the potential for concomitant ocular diseases beyond the macula and the risk of subjects assuming that a negative AMD screening indicates overall good eye health. This might delay adequate assessment by an ophthalmologist and delayed treatment for serious ophthalmic diseases. National guidelines need to be followed and regional differences need to be considered when caring for vulnerable groups. Patients are informed of the research setting they are entering which does not always reflect real-world practice in all participating countries.

Scientifically, the heterogeneity of AMD progression remains a key challenge. Identifying robust biomarkers for accurate AI-based risk prediction remains an unresolved task. Exploration of prognostic biomarkers for AMD progression and endpoints for iAMD is a main study outcome for the SUDETES and APENNINES studies and is currently also being researched through large prospective studies from other groups– including PINNACLE [34] and MACUSTAR [35]. However, these studies focus on a preselected population, i.e. individuals already in clinical experts’ hands which is not fully representative for the real-world community. Additionally, ensuring the generalisability of AI solutions, considering different ethnic and population groups already during the development process, and the transferability of AI solutions to a broad spectrum of OCT devices, is crucial for their implementation in a wide range of healthcare settings [36]. This is a key challenge addressed within I-SCREEN.

A true real-world implementation of AI tools in healthcare remains complex and previous studies highlight the importance of stakeholder engagement at an early stage [27, 37]. Furthermore, while local adoption may be successful, allowing for the innovation to be implemented in different healthcare settings across Europe may pose its challenges. Operating across six countries within and beyond the EU, I-SCREEN must navigate differing healthcare systems, referral pathways, and regulatory environments. A harmonised contractual framework is essential to ensure ethical compliance and data protection across all participating sites.

AI-enhanced diagnostic screening is an opportunity to introduce large scale yet affordable and consistent care for a disease which has become the most frequent cause of irreversible vision loss in the fastest-growing part of the population, older adults. Involving community optometry is a strategy to embed the AMD screening process within the community where this disease occurs in its earliest stages. This is vital given AMD’s nature of early morphological manifestation, but late functional presentation. In addition to the likelihood of significant improvements in patient outcomes, the epidemiological and scientific insight gained from the study design will significantly add to the breakthrough nature of the I-SCREEN approach.

## Summary

### What was known before

- Age-related macular degeneration (AMD) is a leading cause of vision loss, but early stages are frequently underdiagnosed due to minimal or absent visual symptoms.
- Artificial intelligence (AI) has shown to be an efficient tool for detecting and monitoring AMD pathology.
- Several risk factors for AMD progression have been identified, though their role and interactions remain incompletely understood.

### What this study adds

- Introduces a shared care network linking optometrists/opticians with ophthalmologists, creating a novel infrastructure for earlier detection, improved screening, and coordinated management of AMD.
- Establishes the first prospective, real-world dataset of AMD cases collected through community-based care across Europe.
- Develops advanced, data-efficient deep learning methods using longitudinal OCT data to enhance prediction of AMD progression.
- Provides new insights into risk factors driving disease progression.

## Data Availability

Data sharing not applicable to this article as no datasets were generated or analysed. Data generated within the I-SCREEN project will be referenced in all resulting publications in open-access journals. Data access will be granted on a case-by-case basis. In line with requirements for EU-funded projects, the management and sharing of data will adhere to the FAIR (Findable, Accessible, Interoperable, and Reusable) principles.
