# Correction: I-SCREEN: Development of an AI-based infrastructure for community-wide screening and prediction of progression in age-related macular degeneration providing accessible shared care

**DOI:** 10.1038/s41433-026-04771-z

**Published:** 2026-07-23

**Authors:** Marie Louise Enzendorfer, Gregor S. Reiter, Hrvoje Bogunović, Sophie Riedl, Julia Mai, Johannes Schrittwieser, Christoph Stapf, Virginia Mares, Matjaž Mihelčič, Julie-Anne Little, Polona Jaki-Mekjavić, Daniel Barthelmes, Katja Hatz, Javier Zarranz-Ventura, Catherine Creuzot-Garcher, Ruth Hogg, Amir Sadeghipour, Ursula Schmidt-Erfurth

**Affiliations:** 1 Laboratory for Ophthalmic Image Analysis (OPTIMA), Medical University of Vienna, Vienna, Austria; 2 Department of Ophthalmology and Optometry, Medical University of Vienna, Vienna, Austria; 3 Institute of Artificial Intelligence, Center for Medical Data Science, Medical University of Vienna, Vienna, Austria; 4 European Council of Optometry and Optics, Adligenswil, Switzerland; 5 Centre for Optometry and Vision Science, School of Biomedical Sciences, Ulster University, Coleraine, UK; 6 Department of Ophthalmology, University Medical Centre Ljubljana, Ljubljana, Slovenia; 7 Faculty of Medicine, University of Ljubljana, Ljubljana, Slovenia; 8 Jozef Stefan Institute, Ljubljana, Slovenia; 9 Department of Ophthalmology, University Hospital Zurich and University of Zurich, Zurich, Switzerland; 10 Vista Augenklinik Binningen, Binningen, Switzerland; 11 Faculty of Medicine, University of Basel, Basel, Switzerland; 12 Hospital Clínic de Barcelona, Universitat de Barcelona, Barcelona, Spain; 13 Fundació de Recerca Clínic Barcelona-Institut d’Investigacions Biomèdiques August Pi i Sunyer (FCRB-IDIBAPS), Barcelona, Spain; 14 University Bourgogne Europe, Dijon, France; 15 Ophthalmology Department, Dijon University Hospital, Dijon, France; 16 Centre for Public Health, School of Medicine, Dentistry, and Biomedical Science, Queen’s University Belfast, Belfast, UK; 17 RetInSight GmbH, Vienna, Austria; 18 EURICE – European Research and Project Office GmbH, St. Ingbert, Germany; 19 Research and Innovation Services-RISE d.o.o., Zagreb, Croatia

**Keywords:** Medical imaging, Biomarkers

Correction to: *Eye* https://doi.org/10.1038/s41433-026-04487-0, published online 05 May 2026

In this article THE I-SCREEN CONSORTIUM AND COLLABORATORS have been published incorrectly. They should read:

**THE I-SCREEN CONSORTIUM AND COLLABORATORS**

Ursula Schmidt-Erfurth^1,*^, Marie Louise Enzendorfer^1^, Matjaž Turk^1^, Gregor S. Reiter^2^, Johannes Schrittwieser^2^, Sophie Riedl^2^, Julia Mai^2^, Christoph Stapf^2^, Virginia Mares^2^, Klaudia Birner^2^, Anna Eidenberger^2^, Hrvoje Bogunović^3^, Thomas Pinetz^3^, Veit Hucke^3^, Dmitrii Lachinov^3^, Taha Emre^3^, Matjaž Mihelčič^4^, Julie-Anne Little^4^, Fabienne Eckert^4^, Peter Gumpelmayer^4^, Polona Jaki-Mekjavi^6^, Polona Zaletel Benda^6^, Fran Drnovšek^6^, Vlasta Hadalin^6^, Barbara Klemenc^6^, Darko Perovšek^6^, Daniel Barthelmes^9^, Katrin Rudolph^9^, Luca Timoceanu^9^, Katja Hatz^10^, Judith Buck^10^, Charlotte Monnet^10^, Christina Plasencia^10^, Katharina Heck^10^, Javier Zarranz-Ventura^12^, Andrea Mendez-Mourelle^12^, Rafael Castro-Domínguez^12^, Irene Vila^12^, Alicia Pereira^12^, Carolina Bernal-Morales^12^, Lena Giralt^12^, Josep Grau^12^, Valentina Bilbao^12^, Sonia Marías-Perez^12^, Pau Marjalizo^12^, Catherine Creuzot-Garcher^14,15^, Pierre Henry Gabrielle^14,15^, Laure-Anne Steinberg^14,15^, Ruth Hogg^16^, Tunde Peto^16^, Lucia Dalton^16^, Raymond Beirne^16^, Emma McConnell^16^, Deirdre Brannigan-Hogarth^16^, Jill McKeown^16^, Lucy Rundle^16^, Jill Telford^16^, Stephen Vandevyver^16^, Faith Donaldson^16^, Judith Long^16^, Graeme Black^16^, Amir Sadeghipour^17^, Vedran Hrbacek^17^, Ariadne Whitby^17^, Doruk Okcuoglu^17^, Patricia Ambichler^17^, Miriam Lenhart^17^, Sanja Šale^18^, Gabrijela Radić^19^, Lucija Rogina^19^, Slaven Rašković^19^ and Nikolina Lednicki^19^

The original article has been corrected.

